# Intrinsic functional connectivity brain networks mediate effect of age on sociability

**DOI:** 10.1371/journal.pone.0324277

**Published:** 2025-05-28

**Authors:** Yuet Ruh Dan, Savannah K. H. Siew, Junhong Yu

**Affiliations:** School of Social Sciences, Nanyang Technological University, Singapore; University of Maribor, SLOVENIA

## Abstract

Social interaction has been shown to prolong lifespan and healthspan. For older adults living alone, social interaction largely comes from formal social participation, and thus depends on the sociability of the individual. This study aims to understand the effect of age on sociability, and the possible mechanisms behind the change. 196 German participants aged 20–77 (Mage = 37.9) completed a series of questionnaires as part of the Leipzig Study for Mind-Body-Emotion Interactions. Sociability was measured by a subscale of the Trait Emotional Intelligence Questionnaire (Short Form), and network-based statistics were performed on resting-state functional connectivity data to identify networks positively and negatively correlated with age. Mediation analysis was carried out between age and sociability, with both sets of edges as mediators. Overall, age correlated negatively with sociability. The brain network correlating positively with age correlated negatively with sociability, and vice versa for the network correlating negatively with. Both networks independently and completely mediated the age-sociability relationship. The limbic-insular and ventral attention-somatomotor connectivity featured prominently in the age-positive network, while the age-negative network is largely represented by subcortical-parietal and frontoparietal-default mode connections. Networks associated with brain aging can explain the negative relationship between age and sociability. Per Dunbar’s social brain hypothesis, age-related disruption in intrinsic functional connectivity may impair socio-cognitive functions necessary for forming and maintaining relationships, thus causing a decrease in sociability. Psychoeducation of these changes that occur with natural aging could prove useful in the promotion of successful aging.

## 1 Introduction

Recent studies have shown that far from being stable throughout adulthood as was commonly believed, personality is in fact dynamic throughout the lifespan, and is particularly volatile in late adulthood [1]. These changes are represented by the plasticity hypothesis, which states that despite being relatively enduring, personality is dependent on life experiences even into adulthood [2]. As a universal process, aging and its related behaviours are undoubtedly part of a major life experience that can and does lead to changes in personality. For example, aging has been linked to increased agreeableness, as well as decreases in social confidence and enjoyment of social events [3]. Yorkston et al. [4] similarly find sociability and general skills of communication to be impaired in aging, suggesting this change to be due to impairments in speech characteristics as well as declines in physical health and cognition. Following this line of thought, many studies have linked age-related changes in personality to deteriorations in health status [5], such as the relationship between self-reported disabilities and rates of personality change [6]. This could be due in part to a decreasing perception of personal control with increasing health issues.

Sociability is defined as the character trait of being effective in communication, socially assertive and capable at managing emotions [7]. It is crucially linked to social interaction and integration, which in turn are important determinants of health and well-being in old age. Poor social integration can result in effects ranging from poorer psychological well-being to increased risks of cardiovascular disease [8–10]. However, sociability is a personality facet that has also been shown to decrease with age [11], and as such it has been identified as a potential target for the maintenance and promotion of healthy aging. Changes in sociability at different stages in the lifespan and its underlying mechanisms thus warrant further investigation.

In the brain, sociability and its relevant socio-cognitive skills have been generally linked to increased functional connectivity in and between intrinsic brain networks. For example, Killgore et al. [12] found increased connectivity between the limbic network and the default mode network (DMN) to be linked to emotional intelligence, a trait overlapping with sociability in measuring one’s ability to understand and regulate emotions. This relationship was corroborated by Noonan and colleagues [13] who found a correlation between the size of one’s social network and increased connectivity between the DMN and the anterior cingulate cortex (ACC) of the limbic network. Another socio-cognitive skill with implications on emotional management and social abilities is face identity recognition, which has been linked to activation of the ventromedial prefrontal cortex (vmPFC) [14]. Interestingly, Wang et al. [14] suggest that increased social network size was mediated by increased face identity recognition. The implication of the vmPFC in socio-cognitive functioning is further supported by evidence that individuals with autism spectrum disorder have higher regional activation after undergoing social skills training [15]. Greater between-network connectivity of the ventral attention network (VAN), as measured via resting-state EEG, is also implicated in emotional dysregulation [16]. Overall, the DMN, VAN, and limbic structures have been the most strongly correlated with sociability, and heavily implicate changes in functional connectivity of these regions in differing levels of sociability.

Separately, the process of aging also presents with changes in intrinsic brain networks of individuals. Whole-brain rsFC has generally found that aging results in lower within-network connectivity, as well as greater between-network connectivity [17,18] although the latter relationship has been disputed in recent studies [19]. This pattern of network desegregation has been implicated in age-associated declines in processing speed and memory performance [19], as well as reduced cognitive function [20]. Specifically, decreases in functional connectivity within the DMN, VAN, and sensorimotor network are commonly reported in the aging brain. In particular, the DMN connectivity with the rest of the brain has been linked to visual and verbal memory [21] and impaired motor function [18].

Overlaps between the rsFC changes due to age and the rsFC changes observed in relation to sociability are interesting to explore as they may potentially explain sociability changes with age. Regions in the DMN robustly show age-related declines in resting-state connectivity [21] suggesting that functional connectivity changes with age may impair cognitive executive functions and the encompassing socio-cognitive skills (e.g., theory of mind, metacognition) which may alter one’s level of social skills with age [22]. In addition, task-based segregation of the DMN with other brain networks such as the VAN and DAN is shown to be crucial in socio-cognitive tasks [23]. The increase in between-network connectivity characteristic of brain aging and the resultant network desegregation may affect one’s ability to perform socio-cognitive-reliant tasks in daily life and hence affect sociability. The DMN and VAN in functional connectivity-related changes are both implicated in age-related changes and sociability, thus there could potentially be an underlying functional connectivity mechanism linking aging to sociability.

Given the importance of sociability and related skills in health and well-being in later life, it is crucial that we understand the neural mechanisms of sociability with regard to aging. To our knowledge no study has directly examined the relationship between age-related changes in functional connectivity and sociability, despite evidence showing overlaps in rsFC changes linked to both processes. As such, this study aimed to identify the specific brain networks related to age, and further explore if they are able to mediate the relationship between aging and sociability. In this paper, we detail a mediation model of age-related changes in functional connectivity on sociability.

## 2 Methods

### 2.1 Participants

A total sample of 227 healthy German participants was obtained. Participants were aged between 20–77 years old (M_age_ = 37.9, S.D. = 3.7) and there were 82 females (36.1%). Participant data was taken from the Leipzig Study for Mind-Body-Emotion Interactions (LEMON) dataset [24]. Recruitment was performed via public advertisements, leaflets, online advertisements, and information events at the University of Leipzig. Eligibility was determined through a semi-structured telephone interview, and those with a history of cardiovascular disease, psychiatric disease, neurological disorders, malignant disease, positive drug anamnesis or intake of psychopharmacological or chemotherapeutic medication were excluded. Further details on participant data can be found at Babayan et al. [24].

The precise age for each participant was not provided in the dataset due to anonymity reasons, instead the age of each participant is expressed in 5-year bins (e.g., 20–25, 25–30, etc.). For the purpose of the analyses, these 5-year bins are recoded into integer variables (e.g., 20–25 = 1, 25–30 = 2, etc.). Data from 31 participants were also excluded due to data acquisition errors (n = 3), anatomical preprocessing errors (n = 4), functional preprocessing errors (n = 1) and excessive head motion during rsfMRI scans (n = 23), leaving a total of 196 participants of which 67 were female (34.2%). Demographics and mean scores of these participants on the Trait Emotional Intelligence Questionnaire (TEIQue) Sociability subscale as well as NEO Five Factor Inventory (NEO-FFI) personality variables are shown in Table 1. The LEMON study was carried out in accordance with the Declaration of Helsinki and the study protocol was approved by the ethics committee at the medical faculty of the University of Leipzig (reference number 154/13-ff).

**Table 1:** Breakdown of number, sex, TEIQue Sociability subscale scores and NEO-FFI variable scores for participants in each age bin.

		Age Bin								
		20-25	25-30	30-35	35-40	55-60	60-65	65-70	70-75	75-80
Number of participants		78	56	13	1	1	15	18	13	1
Sex breakdown										
	Male	53	43	7	1	1	10	7	6	1
	Female	25	13	6	0	0	5	11	7	0
TEIQue Sociability		5.05	4.84	4.93	4.83	6.33	4.37	4.74	4.65	4.17
NEO-FFI										
	Extraversion	2.48	2.37	2.45	1.42	2.50	2.31	2.42	2.22	2.67
	Neuroticism	1.56	1.53	1.62	2.75	0.92	1.34	1.48	1.31	2.67
	Agreeableness	2.75	2.77	2.79	2.42	3.33	2.73	2.87	2.81	1.33
	Openness for Experiences	2.75	2.71	3.08	3.75	3.17	2.57	2.52	2.37	1.67
	Conscientiousness	2.50	2.52	2.52	1.17	3.25	2.88	3.09	2.97	2.67

*Note.* TEIQue, Trait Emotional Intelligence Questionnaire; NEO-FFI, NEO Five Factor Inventory. Mean scores of each variable for each age bin were calculated and presented.

### 2.2 Procedure

A total of 3 assessment days were conducted as part of the LEMON study: MRI data were taken on the first assessment day, and the psychological assessment measuring sociability was conducted on the second assessment day via computer.

To measure sociability, the *Sociability* subscale of the Trait Emotional Intelligence Questionnaire Short Form (TeiQue-SF) [25] was used. The 6-question subscale measures social awareness, emotional management, communication effectiveness and participation in social situations [26] and has a Cronbach’s alpha of 0.73 [27]. Analysis with item response theory has demonstrated its significance differentiating responders at different levels of sociability, and found the items to have largely high and moderate item information values [7]. This questionnaire is scored on a 7-point Likert scale ranging from 1 (completely disagree) to 7 (completely agree). The higher the score, the higher in sociability a participant is deemed to be. In this dataset, correlational analysis showed a positive correlation between sociability and extraversion, one of the Big Five personality traits from the NEO-Five Factor Inventory questionnaire [28] (*r* = 0.48, p < .001), linking the two traits.

### 2.3 Resting-state fMRI acquisition

Details of fMRI acquisition are taken from Babayan et al. [24]. Magnetic resonance imaging (MRI) was performed on a 3 Tesla scanner (MAGNETOM Verio, Siemens Healthcare GmbH, Erlangen, Germany) equipped with a 32-channel head coil. T1-weighted images were acquired using a Magnetization Prepared 2 Rapid Acquisition Gradient Echoes (MP2RAGE) protocol (TE = 2920ms; TR = 5000ms; TI1 = 700ms; TI2 = 2500ms; FOV = 256mm; 176 sagittal slices; voxel size = 1mm isotropic).

The rsfMRI volumes were acquired using a T2^*^-weighted gradient echo echo planar imaging (EPI) multiband BOLD protocol(TR = 1,400 ms; TE = 30 ms; 64 axial slices; matrix = 88 x 88; voxel size = 2.3mm isotropic) was acquired. The total number of volumes = 657 and the total acquisition time was 15 min 30 s. Participants were instructed to remain awake and lie still with their eyes open while looking at a low-contrast fixation cross. To enable correction for geometric distortions in EPI images from rs-fMRI, a gradient echo fieldmap scan was acquired.

### 2.4 Data pre-processing

The T1 structural images are preprocessed with FreeSurfer 7.2.0 using the default recon-all options. Briefly, this processing includes removal of non-brain tissue using a hybrid watershed/surface deformation procedure [29], automated Talairach transformation, segmentation of the subcortical white matter and deep gray matter volumetric structures (including the hippocampus, amygdala, caudate, putamen, ventricles) [30,31], intensity normalization [32], tessellation of the gray matter white matter boundary, automated topology correction [33,34], and surface deformation following intensity gradients to optimally place the gray/white and gray/cerebrospinal fluid borders at the location where the greatest shift in intensity defines the transition to the other tissue class [35,36]. The resting state fMRI volumes were preprocessed using fMRIPrep 20.2.5 [37]. Functional data were slice time corrected using 3dTshift from AFNI [38] and motion corrected using MCFLIRT [39]. This process was followed by co-registration to the corresponding T1w using boundary-based registration [40] with 9 degrees of freedom, using bbregister from freesurfer. Motion correcting transformations, BOLD-to-T1w transformation and T1w-to-template (MNI) warp were concatenated and applied in a single step using antsApplyTransforms employing Lanczos interpolation.

Subsequently, these preprocessed volumes were denoised by regressing out 6 motion parameters, the average signal of white matter and cerebrospinal fluid masks, global signal, and their derivatives, as well as cosines covering slow time drift frequency band using the load_confounds package (https://github.com/SIMEXP/load_confounds) in python. Scrubbing [41] was carried out to further remove the effects of excessive head motion. The volumes are then smoothed using a 5mm FWHM kernel and subjected to a 0.1Hz low-pass filter. Finally, the brainnetome atlas [42] was used to parcellate the whole brain into 246 anatomical regions corresponding to the nodes of the network and generate rsFC matrices. Participants with excessive head motion, as defined by having more than 20% of their rs-fMRI volumes above the high motion cut-off (relative RMS > 0.25) are excluded from the analyses.

### 2.5 Network-based analysis

Network-based statistics (NBS) using linear models were performed on the rsFC matrices to obtain edges correlated with age. Selection thresholds were set at p < .001 for the edge level, and significant edges were clustered into connected subnetworks. Statistical significance of the networks were then determined by testing network strength against a null-distribution generated by 1000 nonparametric permutations of the data. A threshold of p < .05 was selected at this level.

By interpreting the brain network as a graph, NBS inherently controls the family-wise error rate at the level of subnetworks rather than individual edges. This is particularly suitable for detecting distributed patterns of connectivity differences in large-scale functional connectivity data as weak but significantly interconnected edges are retained, allowing greater sensitivity with preservation of the family-wise error rate in comparison to traditional false discovery correction methods [43,44].

Significant edges that survived edge and network threshold selections were grouped into the age-positive network (APN), corresponding to edges with a positive correlation with age, and the age-negative network (ANN), corresponding to edges with a negative correlation with age. Network strengths for each participant were calculated for both networks by summing up the connectivity strengths of all edges in each network. As such, the greater the overall network score, the greater the connectivity strength of the networks. Analysis was done using the the NBR package [45]. These network strengths were subsequently used for mediation analysis.

### 2.6 Mediation analysis

**Fig 1:**
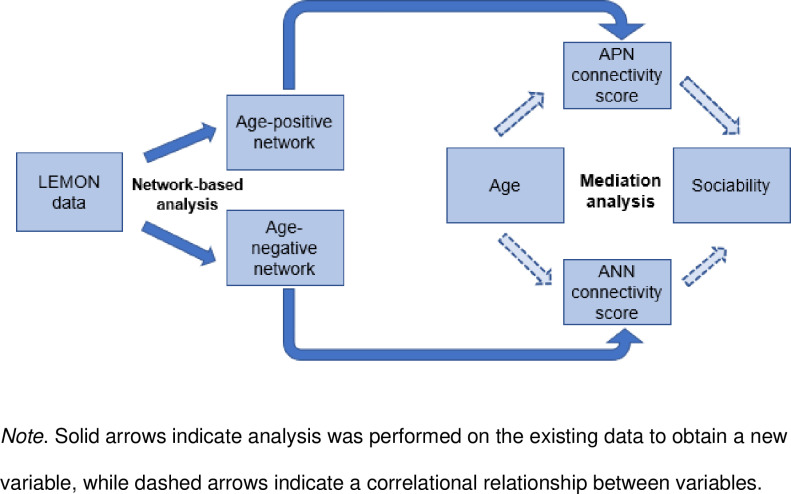
Methodology of data analysis.

Mediation analysis was performed with the APN and ANN scores as individual mediators of the relationship between age and sociability. Analysis was done using R version 4.1.0 [46], with the *mediation* package [47], using quasi-Bayesian confidence intervals with 1000 Monte Carlo simulations. Statistical significance was set at p < .05. The overall pathway of analysis can be seen in Fig 1.

### 2.7 Data analysis

Analysis was performed using R and the *mediation* package [45]. This study’s design and its analysis were not pre-registered.

## 3 Results

### 3.1 Network-based analysis

**Fig 2:**
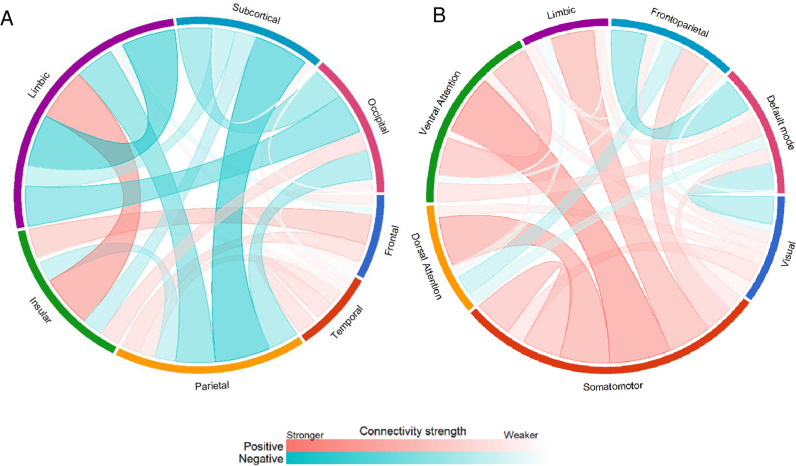
Age-related brain connectivity patterns sorted into (A) brainnetome atlas regions and (B) Yeo’s 7 networks. For the purpose of illustrating the region-wise and network-wise rsFC patterns, the NBS thresholded edges’ regression coefficients are averaged across their respective regions and networks.

The age-related brain connectivity patterns are illustrated in Fig 2. To facilitate understanding of the identified edges and the specific functions that could potentially be implicated in aging, the edges were sorted into the brainnetome atlas regions and 7 brain networks according to Yeo et al. [48]. Of note, the latter does not include subcortical nodes.

Limbic-insular connectivity was identified as the strongest positive correlation within the APN. On the other hand, the subcortical-parietal connectivity and the within-limbic connectivity were identified as the most negatively correlated in the ANN (see Fig 2A). Network-level analysis using the perspective of Yeo’s 7 networks identified edges are shown in Fig 2B. Within the positive connectivity network, ventral attention-somatomotor connectivity was identified as having the strongest correlation with age. Frontoparietal-default mode network connectivity was identified as the most negatively correlated with age.

### 3.2 Mediation analysis

**Fig 3:**
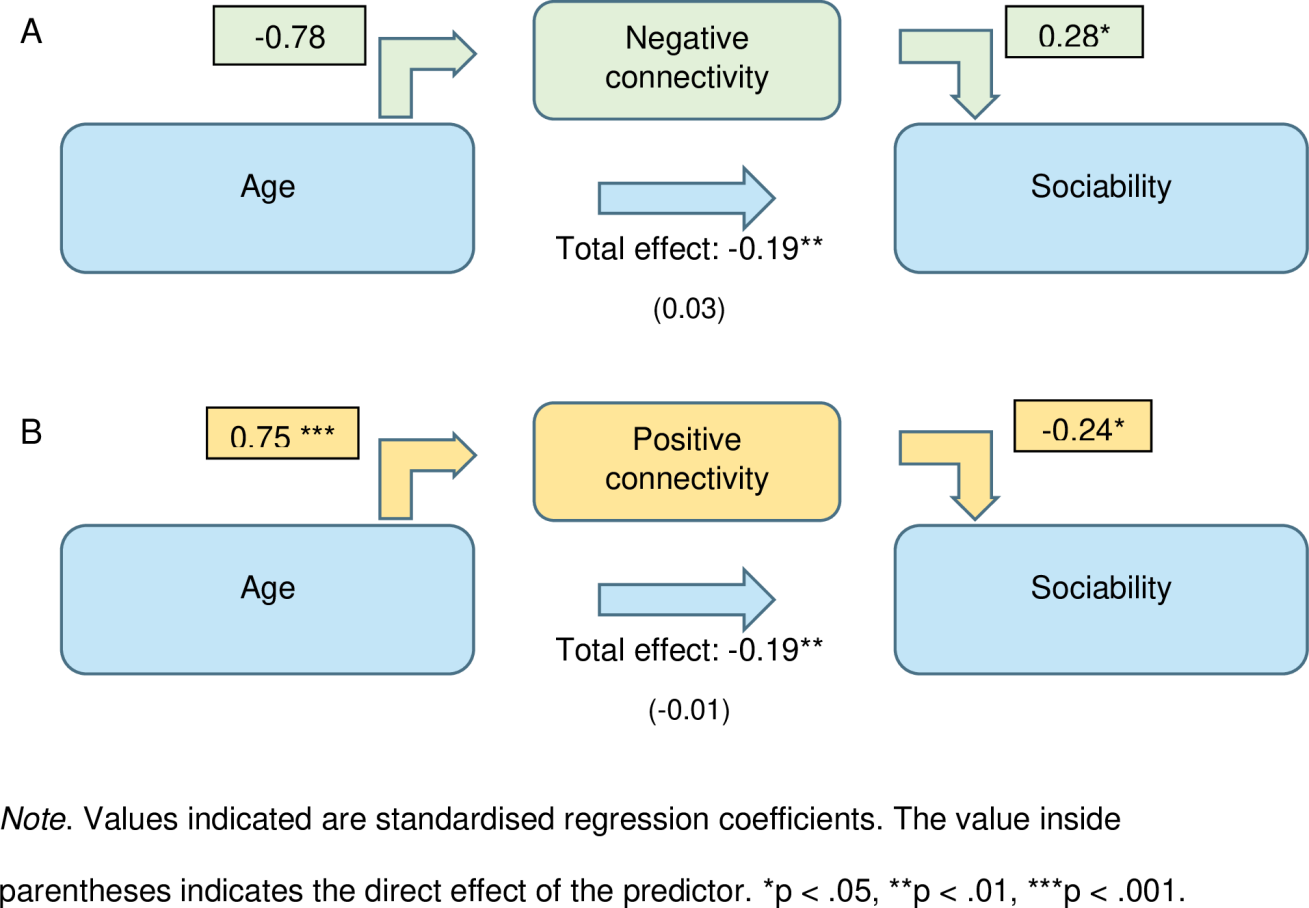
Visualization of mediation models with (A) the ANN and (B) the APN as mediators of each model.

Both the APN (Table 2) and the ANN (Table 3) completely mediated the effect of age on sociability (see Fig 3), with no significant direct effect in both models after controlling for the mediators. In the ANN model, inconsistent mediation was observed [49] as the indirect effects from the mediator were in the opposite valence from the direct effect. This is expected since the sign of the path coefficients are reversed in the ANN model.

**Table 2:** Estimates of the APN-mediated Model of Aging Against Sociability.

	Estimates	95% CI Lower	95% CI Upper
ACME	-0.18 *	-0.34	-0.03
ADE	-0.01	-0.22	0.20
Total Effect	-0.19 **	-0.34	-0.05
Proportion Mediated	0.97 *	0.12	3.80

*Note.* ACME, average causal mediation effect; ADE, average direct effect. *p < .05.

**Table 3:** Estimates of the ANN-mediated Model of Aging Against Sociability.

	Estimates	95% CI Lower	95% CI Upper
ACME	-0.22 *	-0.40	-0.03
ADE	0.03	-0.21	0.26
Total Effect	-0.19 *	-0.33	-0.06
Proportion Mediated	1.19 *	0.12	4.28

*Note.* ACME, average causal mediation effect; ADE, average direct effect. *p < .05 **p < .01.

### 3.3 Supplementary analysis

To ensure statistical robustness of the rsFC results, models were run at p < 0.001, p < 0.01 and p < 0.05, and all significant functional connectivity relationships were preserved. Mediation analyses for all cut-offs remain significant as well. Detailed results of these analyses are presented in the supplementary materials.

## 4 Discussion

In this study, resting-state functional networks positively and negatively correlated with age were identified. The edges in these networks significantly implicated decreased frontoparietal-DMN connectivity, subcortical-parietal connectivity and limbic-limbic connectivity, as well as increased ventral attention-somatomotor connectivity and limbic-insular connectivity. The effect of age on sociability was fully mediated via both networks. Independently, the APN mediated 96.6% of the effect of age on sociability, while the effect of the ANN overrode the direct effect of age on sociability, accounting for 119% of the total effect of age on sociability via an inconsistent mediation.

In the ANN, we identified the connectivity of frontoparietal-DMN networks to be the most negatively correlated with age. This age-related decrease in connectivity is also found in DeSerisy et al. [50], who linked this anticorrelation to reduced cognitive control capacity, as well as the ability to remain focused on tasks. Grady et al. [51] further implicate frontoparietal-DMN connectivity in age-related reductions in associative memory as well as increased negative perceptions of the self. We suggest that decreased connectivity between the frontoparietal and DMN regions may hence be related to impairments in cognition and decreases in self-esteem, thereby resulting in impaired social assertiveness, emotional regulation skills and reduced sociability. Regionally, subcortical-parietal connectivity was also identified as a negatively correlated relationship. Connectivity between these regions has been shown to be involved in language processing as well as selective processing of sensory information which is essential in guiding behavior [52]. Decreased connectivity of these regions could thus be linked to decreased sociability through impaired executive processing.

In the APN, limbic-insular connectivity also showed a significant positive correlation with age, and this relationship has been implicated as part of a social pain network activated in situations of social exclusion [53]. Hyperactivation of this connection with age may thus explain a decreased confidence in social abilities, and lower sociability. However, while no prior studies have directly linked sociability to limbic-insular connectivity, extraversion, which is an often analogous term for sociability, has been associated with greater connectivity between the amygdala and the insular regions [54]. The direction of this relationship is opposite to our results, but is likely due to differential parcellations of the brain in the two studies, as the limbic network in the current study does not take into account subcortical regions such as the amygdala. For example, aside from assertiveness and gregariousness, which are considered aspects of sociability, extraversion additionally measures positive affect and energy. Additionally, vast differences in methodology in collecting resting-state fMRI data [55] could also result in variability of results.

Results of the present study support findings of decreased modularity in the aging brain, exemplified by between-network connectivity increases [17] and within-network connectivity decreases, although only in networks supporting high cognitive functions [56]. This desegregation of brain functional networks has been related to episodic memory [57], executive function [58] and general global cognition [59], which can affect one’s social competencies and therefore propensity for social interaction [60]. As such, a potential explanation for the age-sociability relationship could be an effect of functional dedifferentiation caused by less specialized patterns of functional connections in the aging brain. Such a result would also align with Dunbar’s social brain theory [61]. Dunbar suggests that the size of mammalian species’ neocortices influences their ability to manipulate information about social relationships, which in turn determines the size of their social group. Correspondingly, decreases in functional connectivity between neocortical regions may result in an impairment of socio-cognitive functions necessary for the formation of relationships with others, leading to smaller social networks as one ages.

The current findings are subject to a few limitations. First, the age distribution of the sample was highly skewed towards younger participants, thus our findings on the implications of aging may be less reliable for the older age groups. Furthermore, as mentioned in the methods section, the age of participants was collected in 5-year bins, which may add to noise in our statistical models. In addition, as the participant pool is entirely European, our results may not be fully generalizable to the broader population, especially in light of evidence showing cultural differences in patterns of social interaction and socio-cognitive skills [62]. Third, sociability data was only collected via a self-report questionnaire, which may be subjective and unreliable compared to more objective measures like social network size. Replications of the present study may be carried out with on a population with greater cultural diversity, as well as with more precise demographic data collection. More objective measures of sociability, such as social network analysis or peer-reported measures may also be considered to properly establish sociability, allowing for more comprehensive relationships to be drawn. In addition, the design of this study utilized only cross-sectional data, as longitudinal data was not reported as part of the LEMON study. This limited our ability to generalize findings of the relationship between age and sociability to the process of aging specifically and thus the hypothesized effects of aging on sociability are merely a potential interpretation of the results. Furthermore, as this study is purely correlational, we cannot determine the exact mechanism of this negative relationship between age and sociability that is mediated by changes in functional connectivity networks. Based on the dynamic integration theory of empathy [63], emotional representations are based on basic cognitive representations. As cognitive and biological functions decline naturally with aging, it could implicate emotional schemes and representations, thus resulting in lower empathy and sociability with increasing age [64]. Similarly, personality changes that occur with age could have the potential to alter neural correlates. A study of frontal-lobar degeneration patients showed significant neuroanatomical correlates with different personality trait changes, such as changes in the medial and dorsolateral prefrontal cortex being correlated with reduced extraversion [65]. Reduction in extraversion occurs commonly in aging [66] and such a change could have altered functional connectivity in the brain as well as sociability skills. Rather than aging influencing functional connectivity in the brain resulting in changes in sociability, age-related decreases in sociability could have led to changes in brain functional connectivity, resulting in the functional connectivity alterations observed in the APN and the ANN. In a study conducted by Ibrahim et al. [15], individuals with autism spectrum disorder who underwent social skills training showed higher vmPFC activation, establishing a causal relationship between social skills and functional brain connectivity. Thus, decreases in sociability, potentially due to declines in cognition, could have caused the observed changes in the APN and ANN, as opposed to the connectivity changes being age-related. Undoubtedly, longitudinal studies assessing brain connectivity changes with age and sociability over time would be necessary and useful to ascertain a causal role of the aging process. By clarifying directionality in the relationship between aging, brain connectivity and sociability, longitudinal studies would further have the potential to investigate the effectiveness of interventions related to social interaction for slowing down brain aging. Building on the findings of this current study suggesting that age-related changes in sociability are normal and neuropsychologically driven, a longitudinal analysis would also be well-poised to provide suggestions on novel interventions targeting the functional connectivity networks to address age-related changes in sociability and promote healthy aging as a whole.

Nevertheless, the present study is significant in advancing knowledge on the age-related changes in social behaviors. Evidence from this study suggests that decreases in effective communication, social assertiveness and other relevant social skills measured under sociability do occur normally with increasing age, and these socio-cognitive impairments can negatively influence the size of one’s social network [67]. Coupled with the well-known concept that social isolation at old age is a risk factor for a variety of health conditions ranging from coronary artery disease to Alzheimer’s disease [8,9] and even earlier mortality [10], this finding may potentially be useful for psychoeducation of adults regarding their sociobehavioural changes as they age through their lifespan. By helping to foster greater understanding of themselves and their changing identities, we hope to reduce distress [68] and support the promotion of healthy aging.

## 5 Conclusion

In conclusion, age is correlated with decreased sociability, and this effect is influenced by the age-related changes in intrinsic FC. Network changes with age may be an effect of functional dedifferentiation of the brain, which may impair socio-cognitive abilities that are essential for the formation of social relationships, as per Dunbar’s social brain hypothesis. Psychoeducation of older adults and their caregivers of this natural decline in sociability with age would be useful in encouraging the uptake of successful aging.

## Supporting information

S1 Appendix Functional connectivity networks associated with age: by brainnetome. (DOCX)

S2 Appendix Functional connectivity networks associated with age: by Yeo’s 7 networks. (DOCX)

S3 Appendix Estimates of the APN-mediated model of aging against sociability. (DOCX)

S4 Appendix Estimates of the ANN-mediated model of aging against sociability. (DOCX)
