# Supplementary material for: Intrinsic functional connectivity brain networks mediate effect of age on sociability
Source: PLoS One. 2025 May 28;20(5):e0324277. doi: 10.1371/journal.pone.0324277 (PMC12118820; doi:10.1371/journal.pone.0324277)

S1 Appendix: Functional Connectivity Networks Associated with Age: by brainnetome

Tables of connectivity values

| Edge-level cut-off: p=0.001 | | |  |  |  |  |  |
| --- | --- | --- | --- | --- | --- | --- | --- |
|  | Frontal | Temporal | Parietal | Insular | Limbic | Occipital | Subcortical |
| Frontal | 0.001022 | 0.001522 | 0.005711 | 0.008969 | -0.00233 | 0.000601 | 0.002847 |
| Temporal |  | 0.005548 | 0.004569 | 0.001492 | 0.001574 | 0.001832 | -0.00076 |
| Parietal |  |  | 3.17E-05 | -0.00608 | -0.01148 | -0.01607 | -0.00886 |
| Insular |  |  |  | 0 | 0.015561 | -0.00607 | 0.005811 |
| Limbic |  |  |  |  | -0.01565 | -0.00624 | -0.012 |
| Occipital |  |  |  |  |  | -0.00044 | -0.00997 |
| Subcortical |  |  |  |  |  |  | 0.00039 |
|  |  |  |  |  |  |  |  |
|  |  |  |  |  |  |  |  |
|  |  |  |  |  |  |  |  |
| Edge-level cut-off: p=0.01 | | |  |  |  |  |  |
|  | Frontal | Temporal | Parietal | Insular | Limbic | Occipital | Subcortical |
| Frontal | 0.000607 | 0.00034 | 0.016546 | 0.026463 | -0.0037 | 0.007662 | 0.008838 |
| Temporal |  | 0.017262 | 0.014243 | 0.007101 | 0.012108 | 0.009544 | 0.001293 |
| Parietal |  |  | 0.007789 | -0.0017 | -0.00731 | -0.02525 | -0.00539 |
| Insular |  |  |  | -0.00963 | 0.045606 | -0.02137 | 0.001955 |
| Limbic |  |  |  |  | -0.03686 | -0.0163 | -0.04218 |
| Occipital |  |  |  |  |  | -0.0027 | -0.02021 |
| Subcortical |  |  |  |  |  |  | 0.002868 |
|  |  |  |  |  |  |  |  |
|  |  |  |  |  |  |  |  |
| Edge-level cut-off: p=0.05 | | |  |  |  |  |  |
|  |  |  |  |  |  |  |  |
|  | Frontal | Temporal | Parietal | Insular | Limbic | Occipital | Subcortical |
| Frontal | -0.00221 | -0.00013 | 0.02226 | 0.05194 | -0.00616 | 0.014296 | 0.023273 |
| Temporal |  | 0.037528 | 0.031841 | 0.019911 | 0.01193 | 0.028803 | 0.006794 |
| Parietal |  |  | 0.024395 | 0.011193 | 0.004839 | -0.0266 | 0.001261 |
| Insular |  |  |  | -0.01537 | 0.095504 | -0.04595 | 0.002913 |
| Limbic |  |  |  |  | -0.05532 | -0.01959 | -0.10161 |
| Occipital |  |  |  |  |  | -0.006 | -0.03039 |
| Subcortical |  |  |  |  |  |  | 0.001201 |

Chord diagrams


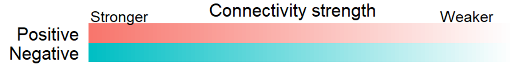
 Edge-level cut-off: p=0.001


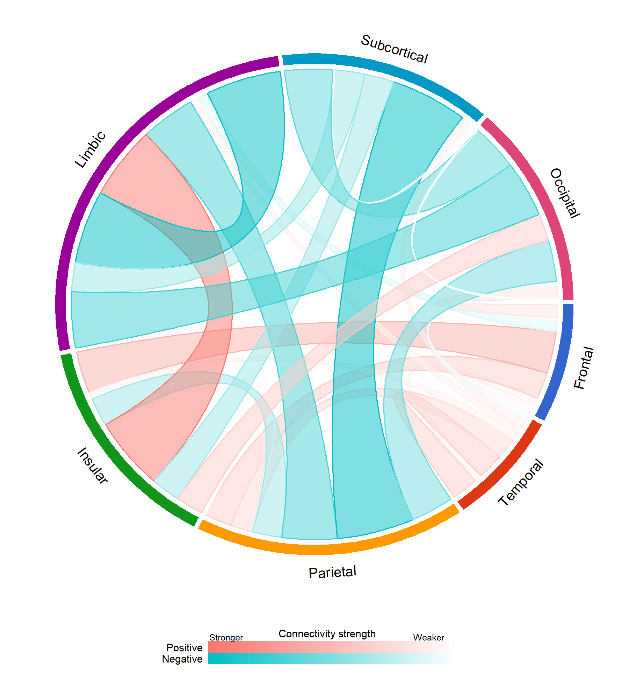


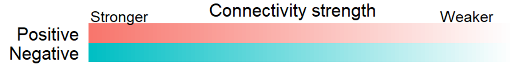
 Edge-level cut-off: p=0.01


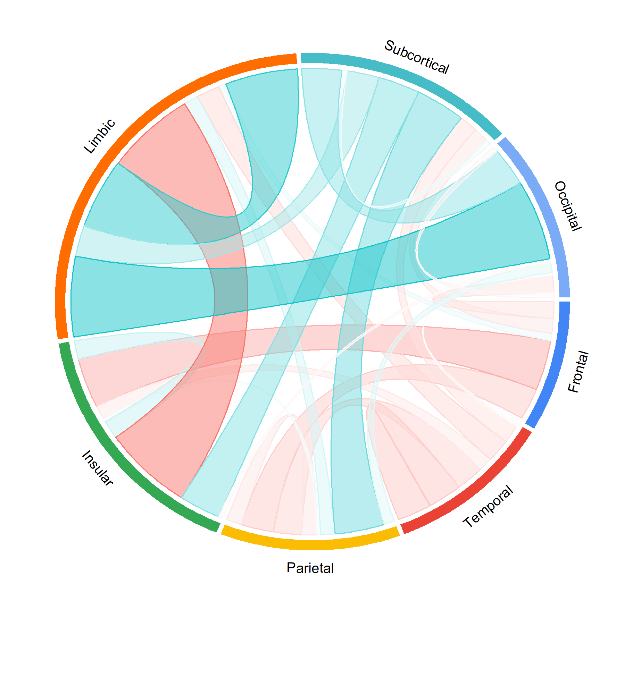


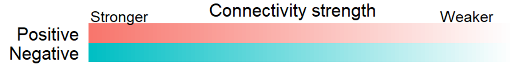
 Edge-level cut-off: p=0.05


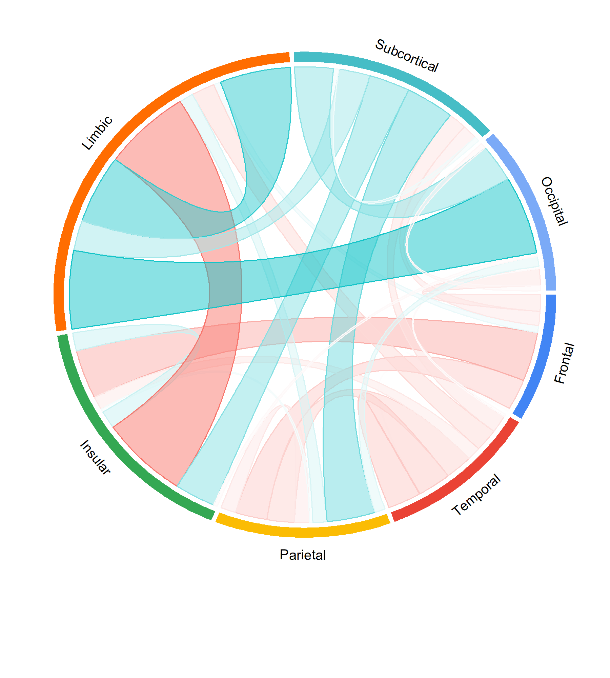

Supplement: S1 Appendix — (DOCX) [file pone.0324277.s001.docx]
