# Supplementary material for: Intrinsic functional connectivity brain networks mediate effect of age on sociability
Source: PLoS One. 2025 May 28;20(5):e0324277. doi: 10.1371/journal.pone.0324277 (PMC12118820; doi:10.1371/journal.pone.0324277)

S2 Appendix: Functional Connectivity Networks Associated with Age: By Yeo’s 7 networks

Table of connectivity values

| Edge-level cut-off: p=0.001 | | |  |  |  |  |  |
| --- | --- | --- | --- | --- | --- | --- | --- |
|  | Visual | Somatomotor | Dorsal attention | Ventral attention | Limbic | Frontotemporal | Default |
| Visual | 0.001464 | 0.003774 | 0.002594 | 0.002259 | 0.002325 | -0.00162 | -0.00566 |
| Somatomotor |  | 0.007887 | 0.010247 | 0.012534 | 0.008987 | 0.006057 | 0.00321 |
| Dorsal attention |  |  | 2.31E-06 | 0.001413 | -0.00107 | -0.00409 | -0.00254 |
| Ventral attention |  |  |  | 0.007896 | -0.00063 | -0.00081 | 0.004011 |
| Limbic |  |  |  |  | -0.00064 | -0.00057 | 0.002105 |
| Frontotemporal |  |  |  |  |  | 0.002324 | -0.00723 |
| Default |  |  |  |  |  |  | -0.00064 |
|  |  |  |  |  |  |  |  |
|  |  |  |  |  |  |  |  |
|  |  |  |  |  |  |  |  |
| Edge-level cut-off: p=0.01 | | | |  |  |  |  |
|  | Visual | Somatomotor | Dorsal attention | Ventral attention | Limbic | Frontotemporal | Default |
| Visual | 0.00292 | 0.018957 | 0.014627 | 0.0019 | 0.010696 | -0.01103 | -0.01591 |
| Somatomotor |  | 0.018308 | 0.028752 | 0.032207 | 0.024991 | 0.00711 | 0.013088 |
| Dorsal attention |  |  | 0.001683 | 0.01955 | 0.005004 | -0.00488 | 0.000462 |
| Ventral attention |  |  |  | 0.015419 | 0.001716 | -0.00207 | 0.012299 |
| Limbic |  |  |  |  | 0.00431 | 0.003909 | 0.012187 |
| Frontotemporal |  |  |  |  |  | 0.004189 | -0.02477 |
| Default |  |  |  |  |  |  | 0.000505 |
|  |  |  |  |  |  |  |  |
|  |  |  |  |  |  |  |  |
|  |  |  |  |  |  |  |  |
| Edge-level cut-off: p=0.05 | | | |  |  |  |  |
|  | Visual | Somatomotor | Dorsal attention | Ventral attention | Limbic | Frontotemporal | Default |
| Visual | 0.000582 | 0.048664 | 0.04143 | 0.005839 | 0.022973 | -0.02323 | -0.02795 |
| Somatomotor |  | 0.037016 | 0.054747 | 0.06109 | 0.052776 | 0.003505 | 0.014818 |
| Dorsal attention |  |  | 0.006102 | 0.046399 | 0.02404 | -0.01118 | 0.010842 |
| Ventral attention |  |  |  | 0.030534 | 0.015925 | -0.00999 | 0.017791 |
| Limbic |  |  |  |  | 0.018021 | 0.00475 | 0.025436 |
| Frontotemporal |  |  |  |  |  | 0.003213 | -0.05912 |
| Default |  |  |  |  |  |  | -0.00234 |

Chord diagrams

Edge-level cut-off:
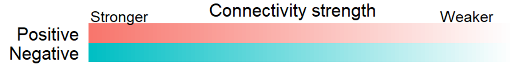
p=0.001


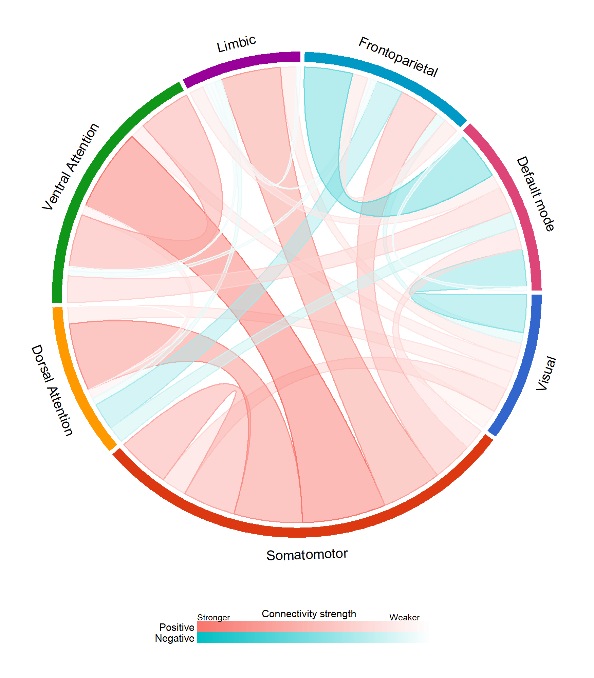


Edge-level cut-off:
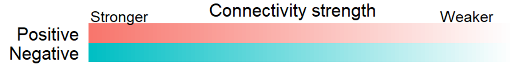
p=0.01


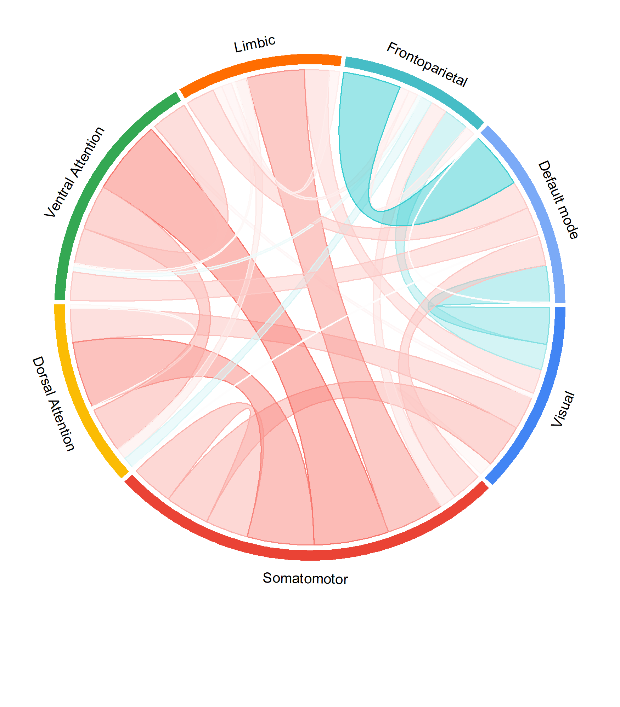


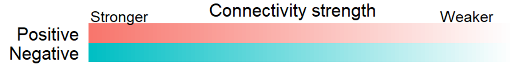
 Edge-level cut-off: p=0.05


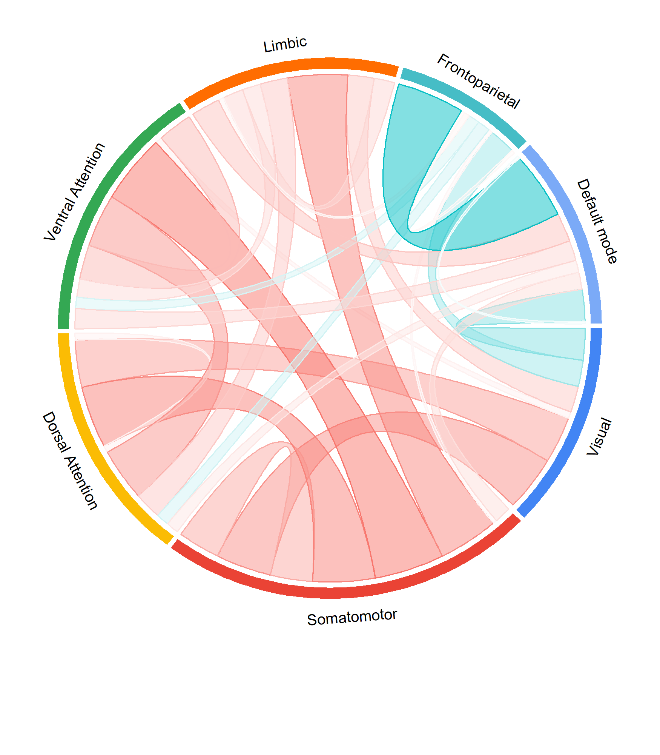

Supplement: S2 Appendix — (DOCX) [file pone.0324277.s002.docx]
