# Supplementary material for: Intrinsic functional connectivity brain networks mediate effect of age on sociability
Source: PLoS One. 2025 May 28;20(5):e0324277. doi: 10.1371/journal.pone.0324277 (PMC12118820; doi:10.1371/journal.pone.0324277)
Supplement: S3 Appendix — (DOCX) [file pone.0324277.s003.docx]

S3 Appendix: Estimates of the APN-mediated Model of Aging Against Sociability

Edge-level cut-off: p=0.001

|  | Estimates | 95% CI Lower | 95% CI Upper |
| --- | --- | --- | --- |
| ACME | -0.18 * | -0.34 | -0.03 |
| ADE | -0.01 | -0.22 | 0.20 |
| Total Effect | -0.19 ** | -0.34 | -0.05 |
| Proportion Mediated | 0.97 * | 0.12 | 3.80 |

*Note.* ACME, average causal mediation effect; ADE, average direct effect. *p < .05 **p < .01.

Edge-level cut-off: p=0.01

|  | Estimates | 95% CI Lower | 95% CI Upper |
| --- | --- | --- | --- |
| ACME | -0.18 * | -0.37 | -0.03 |
| ADE | -0.0042 | -0.22 | 0.24 |
| Total Effect | -0.19 ** | -0.33 | -0.05 |
| Proportion Mediated | 0.96 * | 0.12 | 3.81 |

*Note.* ACME, average causal mediation effect; ADE, average direct effect. *p < .05 **p < .01.

Edge-level cut-off: p=0.05

|  | Estimates | 95% CI Lower | 95% CI Upper |
| --- | --- | --- | --- |
| ACME | -0.20 * | -0.37 | -0.03 |
| ADE | 0.0058 | -0.21 | 0.23 |
| Total Effect | -0.19 ** | -0.32 | -0.05 |
| Proportion Mediated | 1.03 * | 0.10 | 3.83 |

*Note.* ACME, average causal mediation effect; ADE, average direct effect. *p < .05 **p < .01.
