# Supplementary material for: Intrinsic functional connectivity brain networks mediate effect of age on sociability
Source: PLoS One. 2025 May 28;20(5):e0324277. doi: 10.1371/journal.pone.0324277 (PMC12118820; doi:10.1371/journal.pone.0324277)
Supplement: S4 Appendix — (DOCX) [file pone.0324277.s004.docx]

S4 Appendix: Estimates of the ANN-mediated Model of Aging Against Sociability

Edge-level cut-off: p=0.001

|  | Estimates | 95% CI Lower | 95% CI Upper |
| --- | --- | --- | --- |
| ACME | -0.22 * | -0.40 | -0.03 |
| ADE | 0.03 | -0.21 | 0.26 |
| Total Effect | -0.19 * | -0.33 | -0.06 |
| Proportion Mediated | 1.19 * | 0.12 | 4.28 |

*Note.* ACME, average causal mediation effect; ADE, average direct effect. *p < .05

Edge-level cut-off: p=0.01

|  | Estimates | 95% CI Lower | 95% CI Upper |
| --- | --- | --- | --- |
| ACME | -0.24 * | -0.41 | -0.05 |
| ADE | 0.041 | -0.21 | 0.27 |
| Total Effect | -0.19 ** | -0.33 | -0.05 |
| Proportion Mediated | 1.20 * | 0.25 | 4.71 |

*Note.* ACME, average causal mediation effect; ADE, average direct effect. *p < .05 **p < .01.

Edge-level cut-off: p=0.05

|  | Estimates | 95% CI Lower | 95% CI Upper |
| --- | --- | --- | --- |
| ACME | -0.22 ** | -0.41 | -0.05 |
| ADE | 0.033 | -0.19 | 0.27 |
| Total Effect | -0.19 ** | -0.33 | -0.05 |
| Proportion Mediated | 1.14 ** | 0.21 | 5.62 |

*Note.* ACME, average causal mediation effect; ADE, average direct effect. *p < .05 **p < .01.
